# The RISC research project: injury in First Nations communities in British Columbia, Canada

**DOI:** 10.3402/ijch.v72i0.21182

**Published:** 2013-08-05

**Authors:** M. Anne George, Rod McCormick, Chris E. Lalonde, Andrew Jin, Marianna Brussoni

**Affiliations:** 1 University of British Columbia, Vancouver, Canada; 2 Child & Family Research Institute, Vancouver, Canada; 3 University of Victoria, Victoria, Canada

**Keywords:** injury, standardized relative risk, Aboriginal people’s health, Canada

## Abstract

**Background:**

The project, Injury in British Columbia's Aboriginal Communities: Building Capacity while Developing Knowledge, funded by the Canadian Institutes of Health Research (CIHR), aims to expand knowledge on injury rates among First Nations communities in British Columbia (BC), Canada.

**Objective:**

The purpose is to improve understanding of community differences and to identify community-level risk and protective factors. Generally, injury incidence rates in the Aboriginal population in Canada greatly exceed those found in the non-Aboriginal population; however, variability exists between Aboriginal communities, which have important implications for prevention.

**Design:**

This study uses administrative records of deaths, hospitalizations, ambulatory care episodes, and workers’ compensation claims due to injuries to identify communities that have been especially successful in maintaining low rates of injury.

**Results:**

The analysis of risk and protective factors extends the work of Chandler and Lalonde who observed that community efforts to preserve and promote Aboriginal culture and to maintain local control over community life are strongly associated with lower suicide rates.

**Conclusion:**

The discussion on psychological and cultural considerations on healing and reducing the rates of injury expands the work of McCormick on substance use in Aboriginal communities.

Injuries—which for the most part, are preventable—represent a major public health concern. Disturbingly, injury rates are not evenly distributed across different populations. In Canada, injury rates are substantially higher for Status Indians^1^ compared to other residents (1–3). However, these higher rates may mask considerable variability within and between Aboriginal populations. Indeed, Chandler and Lalonde (4,5) examined suicide rates across all First Nations in British Columbia (BC) and showed significant variation between First Nations and between tribal councils, observing an inverse relationship between rates of suicide and the number of protective factors relating to historical links, current governance over services, and progress with lands claims.

## Objective

For this project, injury data from the province of BC were used to examine rates across regions of BC. Further, we propose possible explanations for rate discrepancies between First Nations communities.

## Design

We used the registration database of the province's universal health care insurance program as a population registry. Within this registry, we identified persons as “Aboriginal” if their insurance premiums were paid by the federal government for reason of Aboriginal status, or if their insurance registry records could be linked to a provincial birth or death record indicating Aboriginal status. Subsequently, we linked to our population registry that ascertained aboriginal status to the databases of all people in the province who have been hospitalized, have visited primary care practitioners, or have received workers’ compensation.

We counted hospitalizations from the database of discharge (“separation”) summaries from provincially-funded hospitals. This includes all hospitals providing acute- or rehabilitation-level care, regardless of public or private administration. We included all hospitalizations of residents of the province, regardless of the paying insurance program (provincial, universal, or workers’ compensation). We considered a hospitalization as due to injury if the level of care was “acute” or “rehabilitation” and the most responsible diagnosis on the discharge record was an International Classification of Diseases revision 9 (ICD-9) numeric code in the range 800–999 or an ICD revision 10 (ICD-10) code in the range S00–T98.

We counted visits to primary care from the database of fee-for-service payments to health care practitioners by the province's universal health care insurance program. This database does not include payments made by the workers’ compensation program. We defined an injured visit to a primary care practitioner as a payment for an examination by a general practice physician, emergency physician, nurse practitioner, paediatrician, geriatrician, dentist, or optometrist with an ICD-9 numeric code diagnosis in the range 800–999. This definition excludes payments to surgeons, anaesthesiologists, radiologists, and physical therapists because we consider these to be providers of diagnostic and secondary treatment procedures. Our definition may under-count examinations by emergency physicians and nurse practitioners because these practitioners are often paid salaries or sessional (hourly) fees.

We counted workers’ compensation injuries from the injury file of the province's workplace injury compensation program database system. We did not link to related files to count compensation claims, payments for health care, or wage-loss payments. Hospitalizations due to workers’ compensation injuries would have been counted among hospitalizations described earlier. Some visits to primary care practitioners due to workers’ compensation injuries might have been billed to the province's universal health care insurance system, but this rarely happens because the workers’ compensation program pays practitioners more. We defined a workers’ compensation injury as an injury eligible for workers’ compensation with an ICD-9 numeric code diagnosis in the range 800–999. This definition excludes some chronic conditions eligible for compensation, for example, tendonitis, carpal tunnel syndrome, noise-induced hearing loss, occupational lung diseases, and occupational cancers.

## Results

**Fig. 1:**
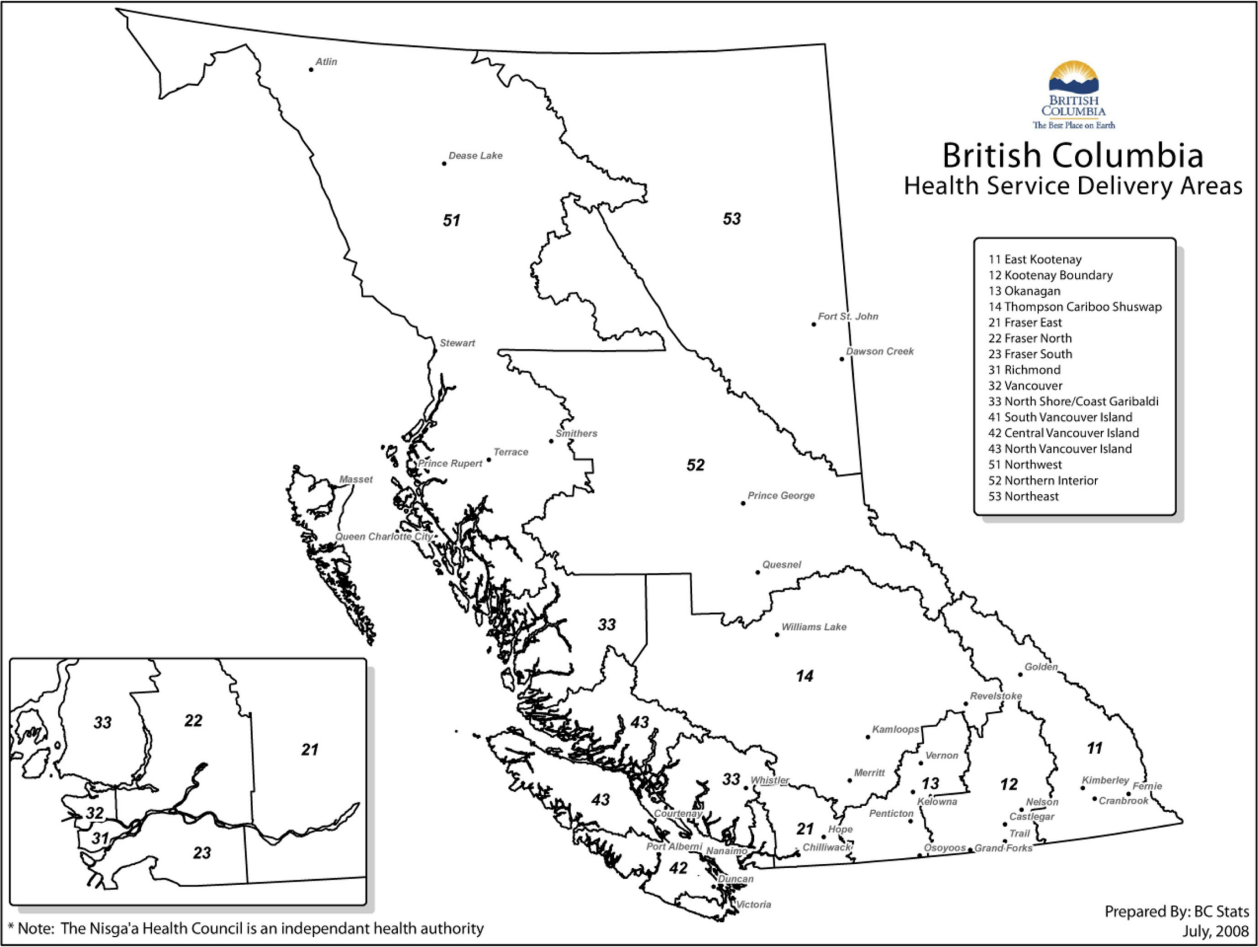
Map of British Columbia, according to health service delivery areas (HSDAs). Map prepared by the government of British Columbia.

**Fig. 2:**
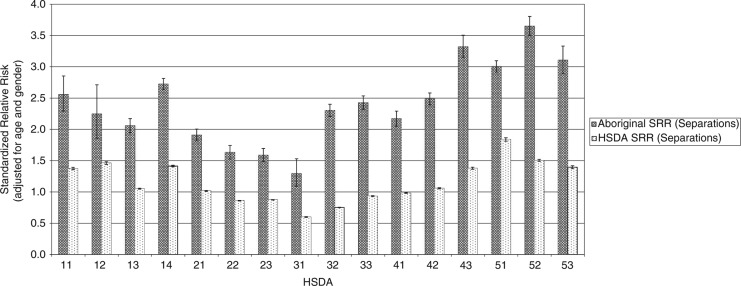
Injury hospitalizations in British Columbia, 1986–2010, by HSDA, by Aboriginal and total injuries.

**Fig. 3:**
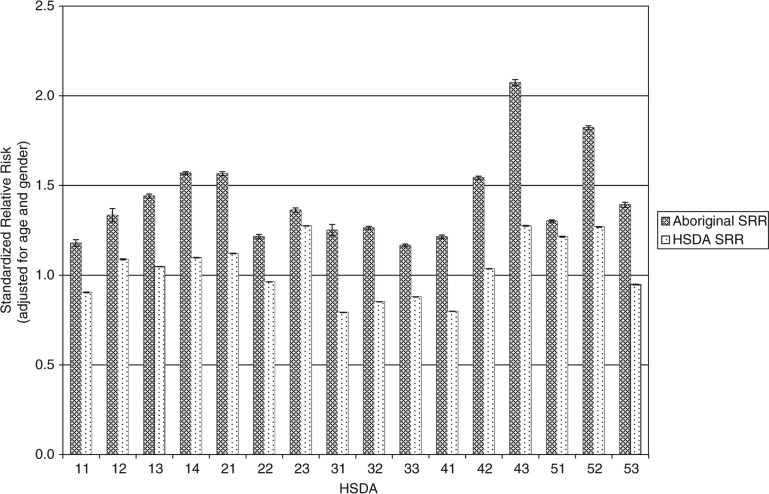
Injured visits to primary care, British Columbia, 1991–2010, by HSDA, total all injuries, by Aboriginal and total injuries.

**Fig. 4:**
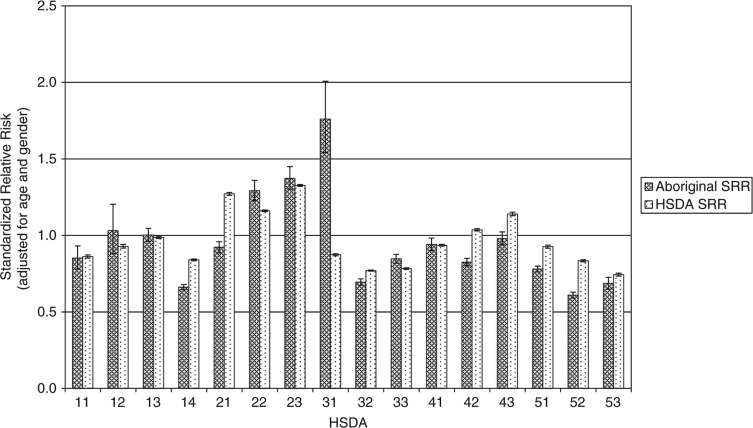
Workers’ compensation injuries, British Columbia, 1986–2009, by HSDA, by Aboriginal and total injuries.

Figure 1 shows the health service delivery areas (HSDAs) of the province. Figures 2–4 show standardized relative risks (SRRs) of hospitalizations attributable to injury (1986–2010), injured visits to primary care providers (1991–2010), and injuries registered for workers’ compensation (1986–2009) among HSDAs of the province. The standardized relative risks have been adjusted for age and gender (indirect standardization method) and are relative to the risk in the total population of BC. The error bars show the 95% confidence intervals for the standardized relative risks. The charts are intended to illustrate that rates of injury (measured by three different, but perhaps overlapping, indices) vary by Aboriginal status and among geographic regions of the province. We advise readers not to make comparisons of hospitalizations, visits to primary care, and workers’ compensation within any specific HSDA because that would be akin to comparing apples to oranges to grapes.

Measured by hospitalizations and visits to primary care, injury risks are consistently higher among the Aboriginal populations than among the total populations of HSDAs. However, there are marked differences in risk among the HSDAs for the Aboriginal populations and for the total populations. In many cases, the 95% confidence intervals do not overlap. This indicates that the differences in risk among HSDAs are likely real and not merely due to random sampling variability. With respect to workers’ compensation injuries, in most HSDAs the injury risk is greater among the total population than among the Aboriginal population, which likely reflects a strong effect of employment rates on risks of this category of injury. We plan more analyses of these data in order to further describe the relationship of geographic, demographic, socioeconomic, and cultural factors to risk of injury.

## Conclusion

In general, injury hospitalizations are highest in the central and northern regions of the province and on northern Vancouver Island for both the Aboriginal and non-Aboriginal populations. For the non-Aboriginal population, the lowest rates of hospitalization are found in and around Vancouver (Vancouver, Richmond, the Fraser Valley; shown as areas 21, 22, 23, 31, and 32 on the map in Fig. 1). This is also true for the Aboriginal population with the notable exception of Vancouver itself, where hospitalization rates are substantially higher for Aboriginal persons.

Injured visits to primary care show a similar pattern with the highest SRR values associated with the central and northern regions as well as northern Vancouver Island for the Aboriginal and non-Aboriginal populations. Lower risk areas for both populations include the largest urban centres of Vancouver and Victoria, as well as the surrounding Fraser Valley.

Workers’ compensation injuries evidence a very different pattern. Risk is highest in the Fraser Valley (areas 21, 22, 23 on the map in Fig. 1) and central (area 42) and northern Vancouver Island (area 43) for the non-Aboriginal population and in the Fraser Valley and south central areas of the province for the Aboriginal population. The exception to this pattern is Richmond (a suburb of Vancouver) that has the highest rate of Aboriginal workers’ compensation injuries in the province but the lowest number of Aboriginal residents in the province—just 0.7% of the total population are Aboriginal according to the most recent census.

## Discussion—psychological, cultural, and spiritual considerations

Descriptions of the impacts of colonization on Aboriginal peoples have been well articulated, including historical trauma, unresolved historical grief, and intergenerational post-traumatic stress disorder (6–9). The government of Canada has acknowledged the tragedy of the federal policy with respect to Aboriginal peoples. In 2008, the prime minister of Canada made a formal apology, stating that (8):

The government now recognizes that the consequences of the Indian Residential Schools policy were profoundly negative and that this policy has had a lasting and damaging impact on Aboriginal culture, heritage, and language. While some former students have spoken positively about their experiences at residential schools, these stories are far overshadowed by tragic accounts of the emotional, physical, and sexual abuse and neglect of helpless children, and their separation from powerless families and communities. The legacy of Indian Residential Schools has contributed to social problems that continue to exist in many communities today …

Resultant social problems such as high rates of alcoholism, substance abuse, and suicide can be linked to intergenerational effects of the residential schools (8). Eduardo and Bonnie Duran point out that once people are assaulted there are psychological effects (7). With loss of power comes despair; the psyche can react by internalizing what appears to be genuine power—the power of the oppressor (7). Self-worth sinks to the level of self-hatred. Self-hatred can be externalized (violence toward others) or internalized (violence toward self). This phenomenon can account for elevated rates of suicide, family violence, substance abuse, and so on—all of which may contribute to higher levels of rates of intentional and unintentional injuries.

## Solutions to reducing injury

Beyond epidemiological studies examining differences in rates of injury, and in particular suicide, few studies provide solutions with respect to preventative initiatives. Chandler and Lalonde (4,5) in their work on suicide, found a relationship between measures of “cultural continuity” and community-level suicide rates. This set of measures includes factors dealing with the past (e.g. proportion of people in the community speaking their traditional language), present (local control over services such as policing or education), and future (e.g. status of land claims). When comparing BC's First Nations, Chandler and Lalonde (4) found that the greater the number of these factors that exist in a community, the lower the suicide rate. Although not causal, their study documents the importance of communities having links to their historical culture, retaining control over their civic lives, and being assured access to their traditional lands and resources.

McCormick (10) found that healing initiatives that involved empowerment, cleansing, balance, discipline/responsibility, and connection/belonging resulted in healing for Aboriginal peoples. In his work describing strategies for Aboriginal people in dealing with substance abuse, for example, McCormick (11) points to many types of connection—to meaning, family, spirituality, and identity and, importantly, to the connection with culture. Disconnection resulted from residential schools and other political policies, racism, trauma, abuse, and other practices. Aboriginal people are developing strategies to deal with the pain of cultural dislocation and the resultant problems.

These early results illustrate the important of using administrative data to geographically illuminate patterns of injuries. The need for further analyses is clear. The next steps of our study will provide data showing the narrowing gap between rates of injury between Aboriginal and non-Aboriginal populations over time as well as differences between Aboriginal communities. We will consider what prevention paths these data illuminate to understand ways to reduce high rates of injury.

## Footnotes

1 Definitions: In Canada, the term First Nations is commonly used to replace “Indian” because many people found the latter term to be offensive, preferring instead to to be called First Nations or First Nations people. Aboriginal peoples and Native peoples are both terms that collectively describe the Indigenous peoples of Canada. Under the Canadian Constitution Act of 1982, three groups are recognized as Aboriginal: Indian peoples, Inuit, and Métis. Three categories apply to Indian peoples: Status Indians, Non-Status Indians, and Treaty Indians. The term First Nations, which applies to both Status and non-Status Indians, is not synonymous with Aboriginal peoples because it does not include Inuit or Métis. Status Indians, who are entitled to be included on the federal government's Indian Register, are recognized as Indians under the Indian Act. This affords them certain rights and benefits. Non-Status Indians consider themselves to be members of a First Nation, but are not entitled to the same rights and benefits because the government of Canada does not recognize them as Indians because they have lost, or unable to prove, their status. Medical care is one of the benefits provided to Status Indians; therefore, only those with Status Indian designation are included in databases such as those used in our analysis. Over the past few decades, First Nation is a term that has replaced the word “band” by many First Nations. Both terms are still used with some preferring “First Nations” in their title and others using the term “band.” For clarity, we use the term First Nation in this paper. A band or First Nation usually consists of one main community or unit of government. In some cases, a band or First Nation remains independent, and in other cases they voluntarily unite to form a tribal council with varying degrees of authority.
